# Tetramethylbenzidine loaded PVA nanofibers strip for visual detection of hydrogen peroxide via Cu^2+^ catalysis and its applications

**DOI:** 10.1186/s13036-026-00632-1

**Published:** 2026-02-24

**Authors:** Marwa A. Aleem, Eman A. Bahgat, Soad S. Abd El-Hay, Samar A. Salim

**Affiliations:** 1 Department of Pharmaceutical Analytical Chemistry, Faculty of Pharmacy, Zagazig University, Zagazig, 44519 Egypt; 2 Nanotechnology Research Centre (NTRC), The British University in Egypt (BUE), El Sherouk City, Suez Desert Road, Cairo, 1183 Egypt

**Keywords:** Hydrogen peroxide, Colorimetric sensor, Polyvinyl alcohol, Tetramethylbenzidine, Electrospinning

## Abstract

**Background:**

Advancements in sensor technology have facilitated the development of cost-effective and highly sensitive sensors which have significantly improved the detection of biological analytes in various fields. This study presents a novel approach for the detection of hydrogen peroxide (H₂O₂), a critical analyte in both biological and environmental systems. A simple and sensitive colorimetric sensing platform was developed based on polyvinyl alcohol (PVA) nanofibers loaded with tetramethylbenzidine (TMB).

**Results:**

The nanofibers were characterized using scanning electron microscopy, confirming the formation of smooth, spindle-shaped fibers with diameters from 324 to 551 nm. The colorimetric detection method utilized TMB, which undergoes oxidation in the presence of H_2_O_2_ catalyzed by Cu^2 +^ resulting in the formation of a characteristic blue color. It revealed a linear increase in absorbance corresponding to H_2_O_2_ concentrations from 3.125 to 100 µM, with a low detection limit of 2.60 µM. The system exhibited excellent specificity and selectivity for H₂O₂ with a visual color gradient scale over common biological interferents such as glutathione, L-cysteine, urea, ascorbic acid, or various ions at a concentration of 100 µM. The new colorimetric sensor was successfully used to determine the H_2_O_2_ level in human urine from healthy subjects demonstrated excellent recovery rates (96–103%).

**Significance:**

This work introduces a simple, cost-effective, and highly selective colorimetric sensor, which is capable of rapid, on-site H₂O₂ detection without reliance on complex instrumentation. Also, the electrospun PVA/TMB exhibits strong potential of nanofibers to be used as a valuable tool for medical diagnostics, environmental monitoring and other biosensing applications.

**Graphical Abstract:**

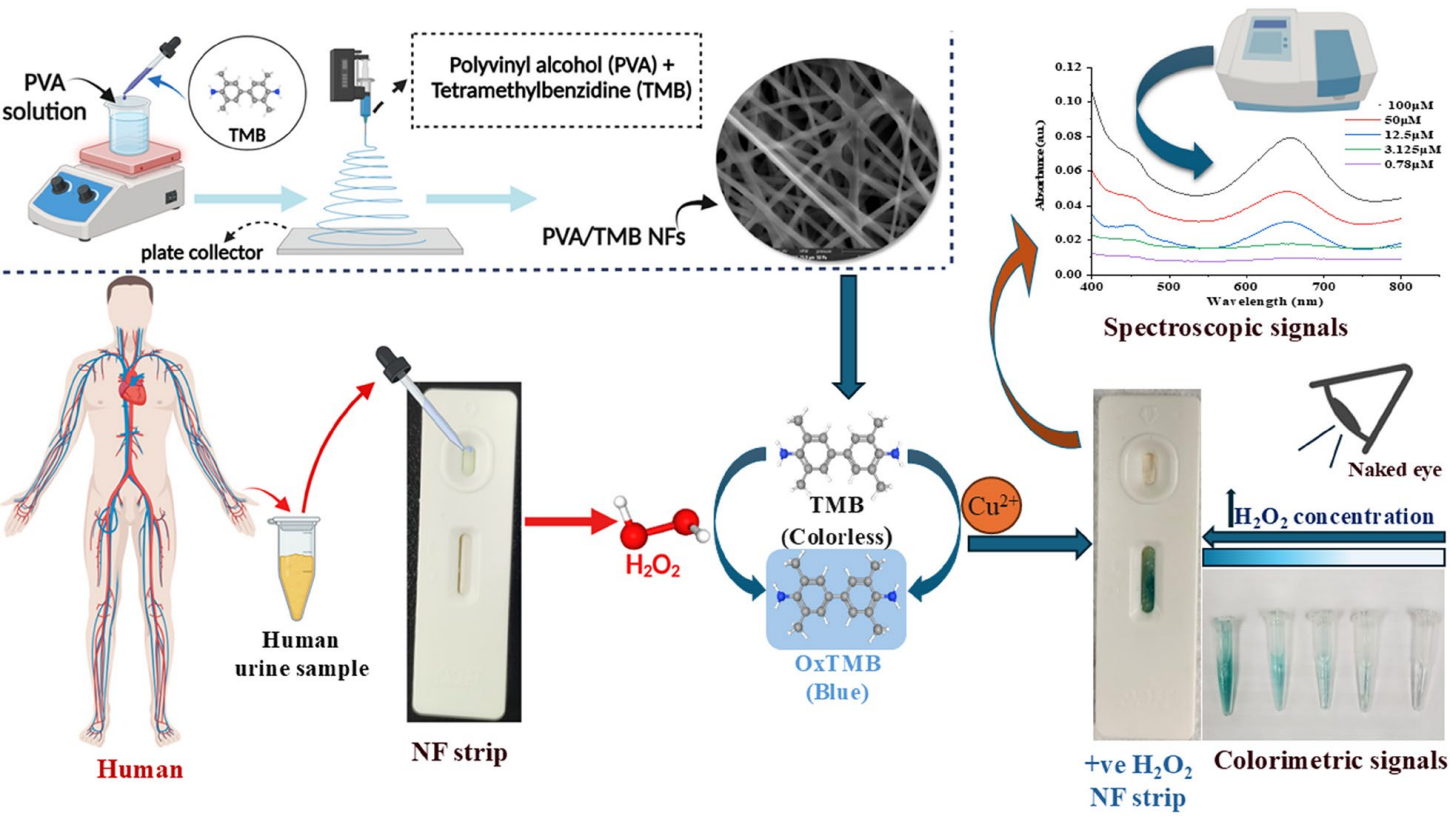

## Introduction

In recent years, the integration of sensors and biosensors has significantly advanced the medical detection and diagnosis of various disorders [1, 2], as well as environmental monitoring [3, 4] and food safety and quality [5]. The increased demand for analytical methods that are rapid, sensitive, selective and cost effective has led to the development of innovative sensing platforms capable of detecting specific analytes with minimal sample preparation without requiring complex techniques. A biosensor consists of a bioreceptor or biorecognition element such as enzymes, antibodies, nucleic acids (aptamer-based biosensors) and a detection system which converts the biological interaction into a measurable electrical or optical signal [1, 6, 7]. Colorimetric sensing approaches which rely on target analyte-induced color changes with naked eye have gained massive attention for both qualitative and quantitative analysis [8, 9]. Colorimetric sensors have been successfully used for detection and identification of a wide range of important analytes, including heavy metals [10], glucose [11] and hydrogen peroxide levels [12].

Recently, electrospun nanofibers (NFs) have attracted great interest due to their unique characterizations such as large surface area, high porosity and functionalization properties [13]. Fabrication of nanofibrous sensors has been used in many fields for detection of various biological analytes and biomarkers [14, 15]. Electrospinning technique is a fascinating method which produces ultrathin fibers from polymer solutions in the range of nanometers in diameter. Polyvinyl alcohol (PVA) is a hydrophilic polymer that has been widely used in fabrication of nanofibers scaffold with porous structures, good physical properties and chemical stability, excellent film-forming and biocompatibility. By utilizing a PVA solution, we can achieve a well-dispersed and uniform formation of nanofibers, enhancing their performance in various applications [16, 17]. PVA nanofiber improves flexibility, provide long-term storage and mechanical strength along with spinning ability [18] as well as PVA serve as an excellent matrix for stabilization of other sensing materials [19] and reagents like TMB. Due to its hydrophilicity, it makes it suitable polymer for construction the nanofiber-based strip [20].

Hydrogen peroxide (H₂O₂) plays a vital role in both biological and industrial systems. In biological systems, it functions in regulating essential physiological processes, including cell and tissue regeneration, growth, proliferation, and migration. They are also widely used in various industrial applications including food manufacturing, paper/textile bleaching, and antiseptic/disinfection [21, 22]. H_2_O_2_ acts as a redox compound which acts as both a powerful oxidant and reductant and there are some target analytes that can give H_2_O_2_ as a main product using oxidase enzymes, e.g., glucose [23], cholesterol [1] and uric acid [24]. H_2_O_2_ displays various essential properties such as oxidizing property, source of energy, gas formation upon decomposition, source of free radicals [25, 26]. Normally, the intracellular concentration of H_2_O_2_ in eukaryotic cells remains at a low concentration (roughly 1–100 nM), but during inflammation and in malignant tumor cells, higher levels exceeding 50 μM can appear in plasma [27]. Subsequently, overproduction of H_2_O_2_ can produce reactive oxygen species (ROS) and strong formation of the hydroxyl radical (·OH) that can induce cellular damage, cytotoxicity, gastrointestinal ulcers and irritation. H_2_O_2_ has been involved as an etiological agent in multiple critical disease conditions including cancer [28], arthritis [29], diabetes, neurodegenerative disorders including Alzheimer’s, Parkinson’s diseases [30], cardiovascular [31], pulmonary diseases, and oxidative stress-related diseases as ageing. Therefore, the accurate determination of concentration of H₂O₂ is vital in pharmaceutical, biological, clinical, and environmental contexts. The reported techniques which were used for its determination such as spectrophotometry, chemiluminescence, fluorimetry [32], colorimetry and electrochemical methods [2, 15] have certain limitations, such as being time-consuming, complex instrumentation, and laborious sample preparation.

To overcome these challenges, this study proposes for the first time a novel, enzyme-free colorimetric sensor composed of PVA nanofibers embedded with tetramethylbenzidine (TMB), a commonly used chromogenic agent due to its noncarcinogenic and non-mutagenic properties using the Cu^2 +^ ions, which act as a peroxidase-mimetic catalyst to facilitate the oxidation of TMB to oxo-TMB in the presence of H₂O₂. A rapid color change from colorless to blue was observed, demonstrating high stability and efficiency with readily available, inexpensive reagents [33]. Copper (Cu) can efficiently catalyze Fenton-like reactions that can react with oxygen or peroxide under mildly acidic and neutral media to generate the highly reactive oxygen species (ROS), such as superoxide anions (O˙_2_^-^) and hydroxyl radicals (·OH). Considering that Cu (II)/H_2_O_2_ species are the significant intermediates for the redox reaction [34]. Hydroxyl radicals (·OH) originated from Fenton reaction between H_2_O_2_ and Cu (II) ions responsible for oxidation of TMB to generate the bluish product. TMB oxidation-induced color development is positively correlated with Cu (II) and ·OH concentrations produced by the Cu-Fenton reaction [35] as explained at the following equations.

$${{\rm{H}}_2}{{\rm{O}}_{2{\rm{ }}}} + {\rm{ }}Cu\left( {II} \right) \to {\rm{ }}Cu\left( I \right) + O_2^{ - } + 2{{\rm{H}}^ + }$$

$${{\rm{H}}_2}{{\rm{O}}_{2{\rm{ }}}} + {\rm{ }}Cu\left( I \right) \to {\rm{ }}Cu\left( {II} \right) + \cdot{\rm{OH}} + {\rm{O}}{{\rm{H}}^ - }$$

$$\cdot{\rm{O}}{{\rm{H}}^{\rm{ }}} + {\rm{ }}TMB \to {\rm{ }}ox\left( {TMB} \right) $$

When compared to other substrates (e.g., phenylenediamine, benzidine, and 2,2′-azino-bis (3-ethyl-benzthiazoline-6-sulfonic acid), TMB displays higher accuracy, sensitivity and precision based on peroxidase detection [36, 37]. The oxidation of TMB with H_2_O_2_ is the base for construction of many colorimetric sensors [37]. The reaction between TMB and H_2_O_2_ is one of the extensively used in colorimetric reactions. The oxidation of TMB by hydroxyl radical (·OH) liberated from the breakdown of H_2_O_2_ in the presence of catalysts such as Horseradish peroxidase (HRP) [11, 38], HRP-like activity as copper acetate monohydrate [24], which is the basis of our proposed colorimetric sensors. In recent years, significant attention has focused on artificial enzyme mimics due to the limitations of natural enzymes. These limitations include a short half-life, high construction costs, stability issues, the need for sophisticated preparation and purification techniques, susceptibility to protease digestion, and denaturation caused by environmental changes. As a result, the practical applications of natural enzymes are often restricted [36, 39]. Artificial enzymes, such as metal nanoparticles (e.g., gold and silver) [10, 37], carbon nanomaterials (e.g., carbon quantum dots) [40] and metal-organic frameworks [41], exhibit peroxidase mimetic. These materials are commonly used due to their exceptional catalytic performance, cost-effectiveness, long-lifespan, and overall suitability for various applications [42]. However, many artificial enzymes investigated have drawbacks, including complex synthesis and the need for multiple processing steps.

This study aims to develop innovative polymer nanofibers strip as a sensitive sensor for the detection of hydrogen peroxide (H₂O₂). The research involves fabricating and characterizing these nanofibers using scanning electron microscopy and electrochemical methods. The effectiveness of colorimetric sensor for detection of H₂O₂ is evaluated under various conditions, including different pH levels and potential biological interferents. Comparative analyses of urine samples from healthy individuals are conducted to assess H₂O₂ levels, correlating findings with existing literature. Ultimately, this study demonstrates the practical applicability of the proposed biosensor for detecting urinary H₂O₂ in individuals, emphasizing its potential for clinical diagnostics and health monitoring.

## Material and methods

### Materials

Polyvinyl alcohol (PVA, (CH_2_CHOH)_n_), Mwt = 72,000 g/mol; 87–89% hydrolyzed), 3,3‘,5,5’-tetramethylbenzidine (TMB), Copper acetate monohydrate (C_4_H_6_CuO_4_·H_2_O) and N, N-Dimethyl-formamide (DMF, ≥99.5%) were supplied by Sigma-Aldrich. L–cysteine (L–Cys) were acquired from techno pharm chem, India. Glutathione (GSH) was purchased from Loba Chemie, India. Ascorbic acid (AA), urea, Sodium chloride (NaCl), Potassium nitrate (KNO_3_), Sodium acetate trihydrate (C_2_H_9_NaO_5_), Sodium hydroxide (NaOH), Glacial acetic acid (CH_3_COOH) and Ammonium acetate (CH_3_COONH_4_) were obtained from Fisher Scientific, U.K. Hydrogen peroxide (30%) obtained from Merck, Germany. Calcium carbonate (CaCO_3_) purchased from Advent, India. The stock solutions of Sodium acetate trihydrate of different pH values were adjusted by acetic acid and NaOH. The solution’s pH was measured with pH meter (Adwa, 11 Hungary).

### Preparation of PVA/TMB nanofibers scaffold

A 10% (w/v) PVA solution prepared by dissolving 10 g of PVA in 100 mL of distilled water under constant stirring at 60 °C overnight. The resulting solution was cooled to room temperature and degassed via sonication. TMB solutions at concentrations of 10, 15, 20, and 40 mM were prepared by dissolving TMB in 5 µL DMF and subsequently blending into the PVA matrix. The mixtures stirred for 2 hours to ensure homogeneous dispersion of TMB. Nanofibers were fabricated by using an electrospinner device (MECC, NANON-01A, Japan) [43]. A plate-type collector covered with aluminum foil at room temperature. The solutions loaded into 6 mL plastic syringes fitted with 22 G stainless steel needles and connected to a PTFE tube. Electrospinning was conducted at room temperature 25 °C and 40% relative humidity with an applied voltage of 21–22 kV and a tip-to-collector distance of 15 cm as aligned in Table 1. The collected nanofibers were dried under vacuum and stored in a vacuum desiccator prior to performing various characterizations and exploring further applications.

**Table 1:** The compositions and electrospinning condition of nanofibers scaffolds

Scaffold solution	Concentrations		Spinning condition		
	PVA(w/v)	TMB (mM)	Applied Voltage(kv)	Feed rate(mL/ hr.)	Physical observation
PVA	10%	-	21	0.5	Good and smooth nanofibers
PVA/TMB-1	10%	10	22	0.5	Uniform beads-free nanofibers
PVA/TMB-2	10%	15	22	0.4	Beads-free nanofibers
PVA/TMB-3	10%	20	22	0.5	Uniform and continuous nanofibers
PVA/TMB-4	10%	40	21	0.3	Good forming nanofibers

### Instrumental characterization of PVA/TMB NFs scaffolds

**Fourier Transform Infrared Spectroscopy (FTIR):** Spectra were recorded using a Bruker Vertex 70 spectrometer in the range 4000–400 cm^− 1^ in transmittance mode [44, 45].

**X-ray Diffraction (XRD):** X-ray spectra were collected using (Malvern PANalytical, England, UK), using a tube with a copper anode radiation. The diffraction patterns were acquired in the 2θ scan range of 5–80° with a step size of 0.02° and at the rate of 0.2 s per step [46].

**Scanning electron microscopy (SEM):** Field emission environmental scanning electron microscope (FE-SEM) model (Quattro S, Thermo Scientific USA) was conducted to examine the surface morphology and size of nanofiber scaffolds. Each electrospun nanofiber was cut into a piece of 2 cm × 2 cm. The average diameter of various mats was quantitatively estimated using Image J software by studying and analyzing 60 random nanofibers.

**UV-Visible Spectroscopy:** A Cary 5000 spectrophotometer from Thermo-Fisher Scientific, USA. Used to record spectra. A characteristic absorption peak of oxidized TMB at 655 nm was employed for qualitative and quantitative investigation. All measurements were carried out at room temperature.

### Peroxidase-like catalytic activity of Cu^2+^ solution

The catalytic potential of the Cu^2 +^ -mediated PVA/TMB NFs toward hydrogen peroxide was investigated through colorimetric response measurements [24, 47]. In Eppendorf, cut a piece of PVA/TMB NFs which acted as peroxidase substrate, add 5 μL Cu^2+^ (40 mM) catalyst solution. Then 10 μL of H_2_O_2_ (100 μM) was added. The UV-visible absorbance spectrum of the oxidized product displayed a pronounced peak at 655 nm and reflected the catalytic activity of Cu^2+^ solution.

### Detection of H₂O₂ by PVA/TMB NFs

A colorimetric method was developed to determine hydrogen peroxide [48]. Achieved by cutting six equal square parts of nanofiber sheets from each scaffold and putting them into folded filter papers which act as adsorbed pads, then 5 μL of Cu^2+^ solution was added to each piece of nanofiber followed by 10 μL of aqueous solution with varied concentrations of hydrogen peroxide (100, 50, 12.5, 3.125 and 0.78 μM) added separately for about 30 min. The intensity of the resulting color was visually observed, The PVA/TMB NFs test strip showed clearly visible color changes from white to blue. The colorimetric signals can be observed by taking photos with a smart mobile phone and measuring at 655 nm.

### The Effect of pH

The pH of the buffer solution has been shown to have a significant impact on the catalytic activity of peroxidases [47]. thus, the sensor’s response was evaluated over a pH range of 4.0–10. In an Eppendorf tube containing a square piece of PVA/TMB NFs, 5 µL of Cu^2+^ ions (40 mM) was added to acetate buffer solution with varied pH values. following the addition of 10 µL of a H₂O₂ (100 µM), incubated for 5 mins, then measured at 655 nm. The stock solution of Cu^2+^ ions was prepared using various pH levels of (4.0, 7.0 and 10.0) acetate buffer solutions. The pH values of the various buffers adjusted by using a pH meter.

### Evaluation of stability PVA/TMB NFs over time

The stability of the produced nanofibers was assessed by maintaining the strip in a dark and cool place for a period of 12 months at room temperature, then measuring its performance. Five batches of PVA/TMB NFs were prepared from promising result of sheets (40 mM of TMB) at preparation (as mentioned before) and after 12 months for reproducibility and stability study. PVA/TMB NFs cut into 2 × 2 sq. cm and putting them on folded filter paper act as absorbed pad, in the same condition of the test of H_2_O_2_ mentioned before, then 5 μL of Cu^2+^ solution added to each piece of prepared scaffold followed by 10 μL of aqueous solution with varied concentrations of H_2_O_2_ (100, 50, 12.5, 3.125 and 0.78 μM) was added separately. The stability was monitored by all samples collected at regular intervals of 0 and 12 months, recorded the time and color change by naked eye and the absorbance spectra at 655 nm.

### Interference study

To evaluate the specificity of the prepared PVA/TMB NFs towards H_2_O_2_, we choose nanofibrous mats with highest concentrations of TMB (20 and 40 mM) [47, 48]. We have studied the potential interference of substances that can coexist in human urine such as GSH, L-Cys, urea, AA and 4 types of ions (Na^+^, K^+^, NH^+^ and Ca^2+^). The tested concentrations 10 µM for H_2_O_2_ and 100 µM for other interferants (Ten times the concentration of H_2_O_2_). All experiments were carried out under the same condition where the H_2_O_2_ was tested. The mentioned interferants were separately added into the Eppendorf which containing PVA/TMB NFs, then 5 µL of 40 mM of Cu^2+^ was added for about 10 mins. The visual color changes were measured at 655 nm and photographs were captured.

### Measurement of H_2_O_2_ in real samples

To preserve donor confidentiality, our research team used stringent anonymization processes on a human urine sample obtained from two healthy adult donors. To preserve sample integrity, immediately after collection, refrigerated at 4 °C. The specimens centrifuged at 4000 rpm for 30 minutes to remove residues and collect the supernatant for analysis, diluted by 10-fold with deionized water [49]. And then various amounts of H_2_O_2_ spiked to urine samples to assess the suggested method’s accuracy. Finally, samples were analyzed in triplicate using the same colorimetric and spectrophotometric methods described above.

## Results and discussion

### Fabrication and compositions of PVA/TMB NFs

To optimize the morphology of electrospun polyvinyl alcohol/tetramethylbenzidine (PVA/TMB) NFs, various fabrication conditions were studied, with a focus on generating uniform, bead-free fibers. Nanofibrous scaffolds were produced using 10% w/v PVA solutions with different concentrations of TMB (10, 15, 20, and 40 mM) and electrospun at 22 kV with a consistent distance of 15 cm between the spinneret syringe needle and the aluminum foil collector. The selected NF scaffolds were determined based on physical observations, Table 1 revealed that PVA produced well-formed smooth nanofibers, while the addition of TMB resulted in dense, continuous fibers with a homogeneous and aligned distribution. One of the critical parameters affecting the topography of the formed nanofibers was the feeding rate of the polymer solution from the syringe needle. Uniform nanofibrous scaffolds were successfully fabricated at feeding rates of 0.4 to 0.5 ml/h. However, at higher TMB concentrations (40 mM), a reduced feeding rate of 0.3 ml/h was necessary. Additionally, using TMB concentrations greater than 40 mM did not yield fibers with the desired characteristics. After 4 hours of electrospinning, the resulting PVA/TMB nanofibers exhibited uniform, stretchable, and bead-free characteristics, demonstrating the highest TMB loading.

### FT-IR spectra of nanofibrous scaffolds

**Fig. 1:**
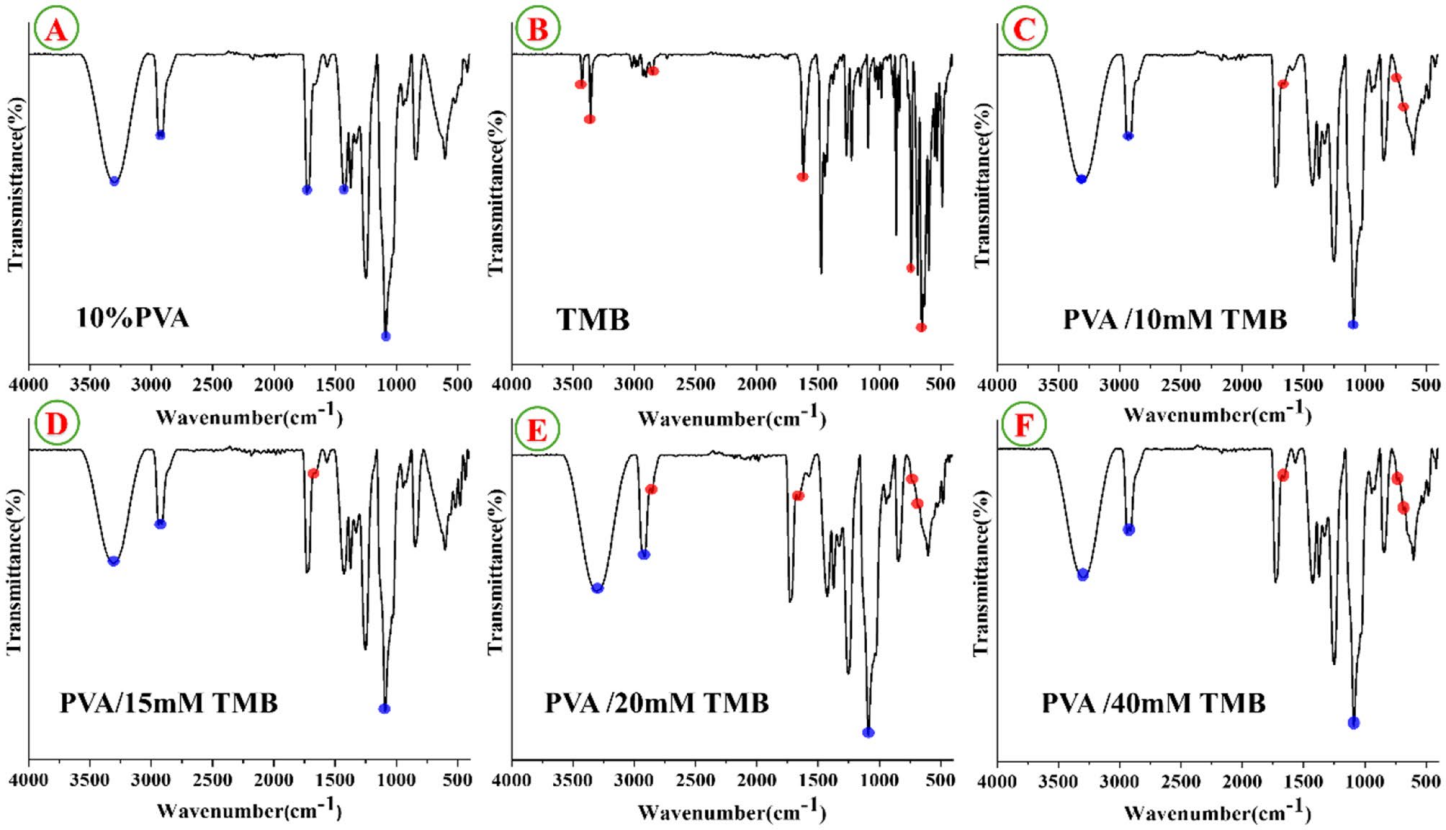
FT-IR spectra of (**A**) PVA, (**B**) tetramethylbenzidine (TMB), (**C**) PVA/10 mM TMB, (**D**) PVA/15 mM TMB, (**E**) PVA/20 mM TMB and (**F**) PVA/40 mM TMB. The main characteristic peaks of each spectrum are highlighted to display changes in formed nanofibers

The spectrum in (Fig. 1A)

, shows the absorbance peaks of pure PVA nanofibers presented at 3302 cm^−1^ (O-H stretching) from the intermolecular and intramolecular hydrogen bonds, 2939 cm^−1^ (H-C-H stretching of alkyl group), 1730 cm^−1^ carbonyl stretching bond (C = O), 1425 cm^−1^ (CH stretching) and 1085 cm^−1^ (C-O-C stretching) of acetyl group, respectively [50–52]. In Fig. 1Bthe pure TMB reveals the characteristic vibration peaks at 3500–3300 cm^−1^ band corresponded to -NH vibrations (amino group) [53]. These noticed vibrations in TMB disappeared in all FT-IR spectra of PVA/TMB NFs, indicating the TMB interacted and encapsulated with PVA through amino group (-NH_2_). The characteristic vibration peak located at 1660 cm^−1^ which related to stretching aromatic rings were noticed in all TMB loaded PVA NFs as shown in Fig. 1C, D, E and F. The scaffold loaded with (20 mM) TMB showed introduction to a new peak at 2855 cm^−1^ that corresponding to (N-H stretching) as observed in Fig. 1E. Moreover, the scaffolds loaded with high concentration of TMB (20 and 40 mM) showed the presence of two new peaks at lower frequency at 735 and 685 cm^−1^ revealed the C-H di-substitution to benzene ring as represented in Fig. 1E and F. These observations assign that TMB was successfully embedded and capsulated into PVA NFs.

### XRD analysis

**Fig. 2:**
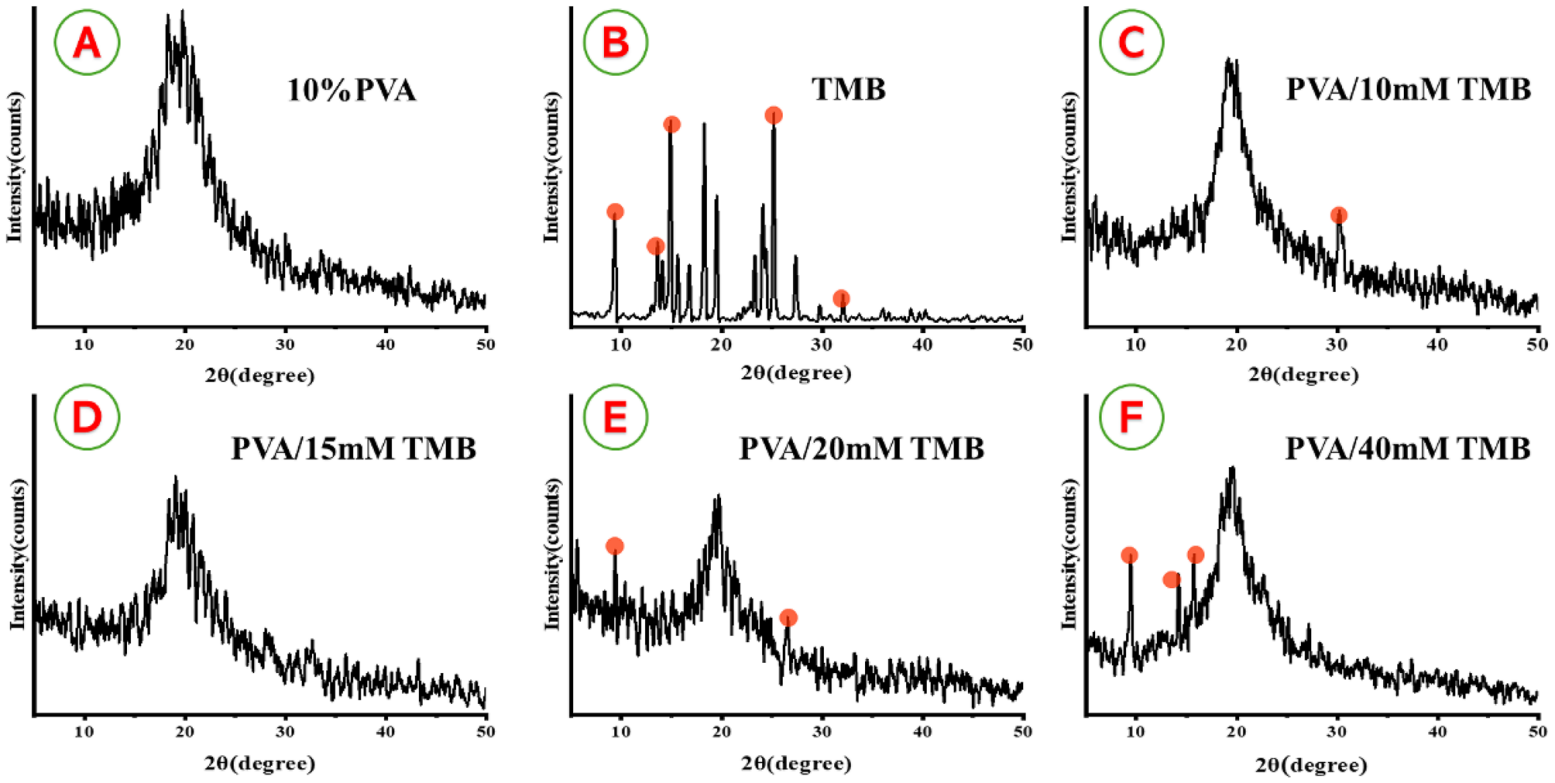
XRD patterns of (**A**) PVA NFs, (**B**) tetramethylbenzidine (TMB), (**C**) PVA/10 mM TMB, (**D**) PVA/15 mM TMB, (**E**) PVA/20 mM TMB and (**F**) PVA/40 mM TMB. The main characteristic peaks related to loaded TMB of each spectrum are highlighted to display changes in the formed nanofibers

XRD was employed to assess crystallinity of the nanofibrous scaffolds. The pure PVA NFs exhibited a broad peak around 2θ = 19.5° [17, 51, 54], as illustrated in Fig. 2A. In contrast, the pure TMB powder diffraction patterns revealed main peaks at approximately 2θ values of 9.37°, 13.63°, 14.93°, 15.61°, 19.49°, and 32.03°, indicating the crystalline structure of TMB (Fig. 2B). International Centre for Diffraction Data (ICDD) standard data was compared to the peak positions in the diffraction pattern, specifically card No. 00–042-1739 and card No. 00–033-1902. All reflections matched well with the ICDD patterns, confirming the crystalline nature of TMB. The electrospinning technique was found to decrease the crystallinity of TMB. XRD analysis further demonstrated how the crystallinity of TMB could be influenced by its incorporation into an amorphous structure when loaded onto PVA at varying concentrations (10, 15, 20, and 40 mM). At the lowest concentration of TMB (10 mM) in PVA/TMB NFs, a peak like that of pure PVA NFs was observed, along with the introduction of a new peak at 2θ = 30.15°, corresponding to the integration of TMB (Fig. 2C). However, at concentrations of 15 mM and 20 mM of TMB, no sharp bands attributed to crystalline TMB were observed in the diffractograms (Fig. 2D and E). At the highest concentration of TMB (40 mM), the PVA/TMB NFs displayed three sharp peaks at 2θ values of 9.43°, 14.19°, and 15.71°. These observations indicate a semi-complete conversion of TMB into an amorphous state, uniformly integrated within the PVA NFs (Fig. 2F).

### Investigation of nanofibrous mats morphology

**Fig. 3:**
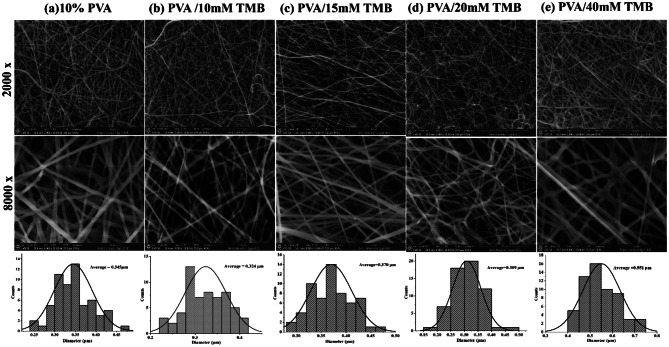
SEM images of electrospun for (**a**) pure PVA NFs, (**b**–**e**) PVA/TMB NF mats at various TMB concentrations (10, 15, 20 and 40 mM), and nanofiber diameter distribution histograms, respectively. (original magnification 2000× and 8000×; scale bar = 10 µm)

The SEM images and histograms of electrospun PVA NFs with varying amounts of TMB (10, 15, 20, and 40 mM) are presented in Fig. 3. The results indicate that pure PVA NFs are uniformly and randomly oriented, exhibiting no beads or fiber bundles, with a consistent diameter distribution centered around 345 nm (Fig. 3a) [17, 23, 55]. Upon incorporating TMB at different concentrations, the PVA/TMB NFs remained largely unchanged, retaining their bead-free characteristics. The average diameters and diameter distributions varied, measuring approximately 324 nm, 370 nm, and 309 nm for TMB concentrations of (10, 15 and 20 mM), respectively (Fig. 3b, c, and d). However, increasing the TMB concentration to 40 mM resulted in a notable expansion in fiber diameter, reaching up to 551 nm (Fig. 3e). All nanofibers displayed uniform distribution and smooth surfaces. The increase in fiber diameter is attributed to the heightened the polymer solution’s viscosity, as previously reported by Hussein et al. (2021) [56]. This demonstrates that the diameter of PVA nanofibers increases with higher TMB concentrations. Notably, no TMB crystals were seen on the nanofibers’ surface or in the surrounding areas, indicating that TMB was completely encapsulated and uniformly loaded onto the PVA NFs scaffold.

### Peroxidase-like activity

**Fig. 4:**
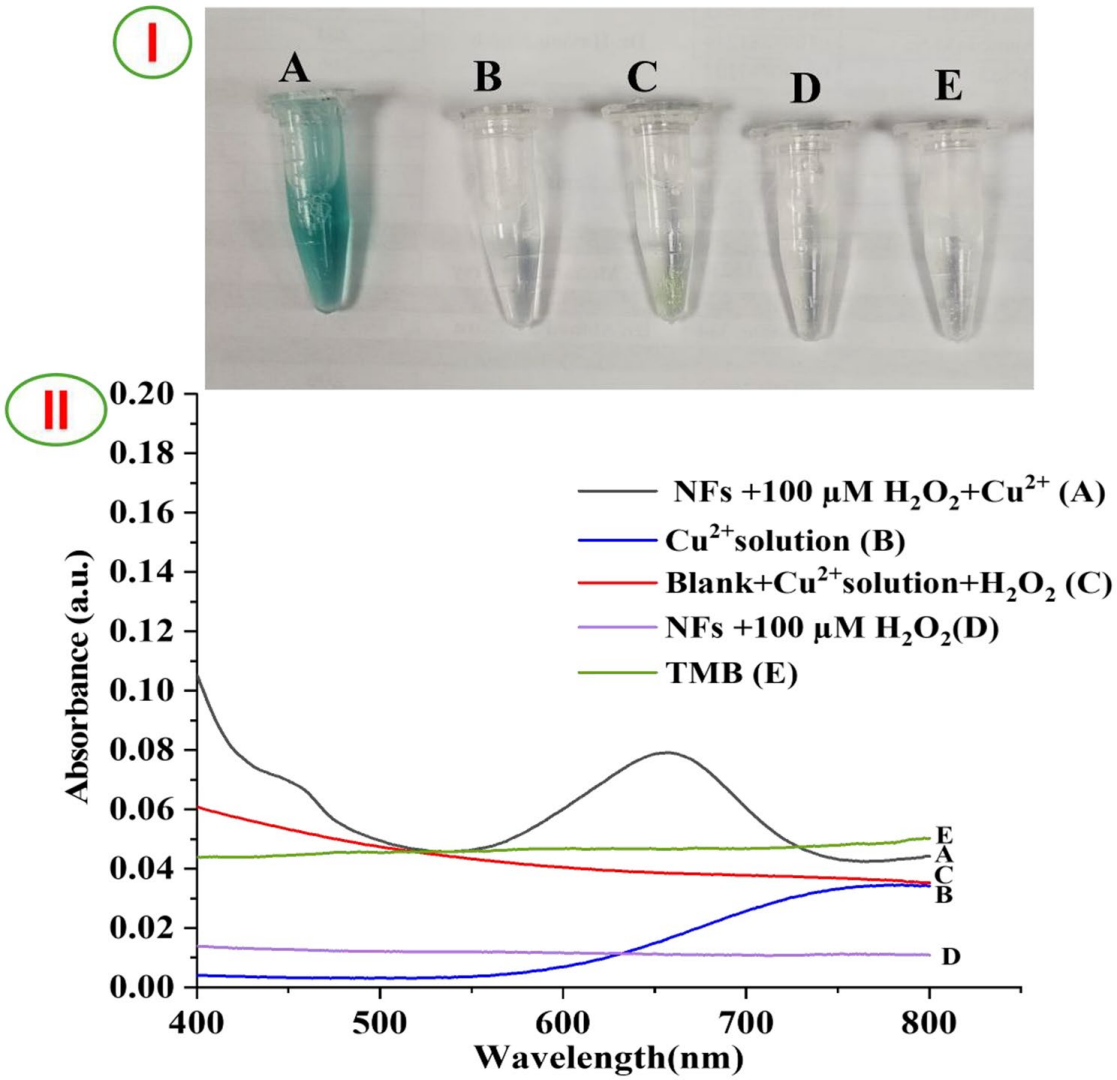
(**I**) optical photographs of observed color, (**II**) UV–vis spectra of (**A**) PVA/TMB NFs+ Cu^2+^ catalyst +100 µM H_2_O_2_, (**B**) 40 mM Cu^2+^ solution, (**C**) PVA NFs + 40 mM Cu^2+^ solution +100 µM H_2_O_2_, (**D**) PVA/TMB NFs+ 100 µM H_2_O_2_ and (**E**) TMB solution

As we explained above, For the oxidation of peroxidase substrate, Cu^2+^ solution makes an excellent peroxidase mimic. In this instance, Cu^2+^ solution exhibits significant peroxidase-like activity towards TMB oxidation in the presence of H_2_O_2_ [24, 57]. As shown in Fig. 4, the catalytic process can be monitored by UV-Vis absorption spectrum and visualized by naked eye. It is discovered that when the Cu^2+^ solution is added, TMB turns blue in the presence of H_2_O_2_. Accordingly, Fig. 4A shows a characteristic absorption peak at 655 nm, indicating that the interaction of PVA/TMB NFs with H_2_O_2_ was enhanced by Cu^2+^. In contrast, when PVA/TMB NFs were mixed with Cu^2+^solution or PVA NFs (as a blank) with 40 mM Cu^2+^solution or blank with 100 µM H_2_O_2_ no blue color change and no absorbance 655 nm of oxidized TMB was observed (curve B and C). Interestingly, in contrast to the absorption spectrum of 100 µM H_2_O_2_ with TMB and TMB solution (curve D and E) no clear peak was observed at 655 nm. In the absence of any TMB, H_2_O_2_ or Cu^2+^ solution, there won’t be any detectable absorption peak at 655 nm. During the catalytic reaction H_2_O_2_ molecules activated by Cu^2+^ to produce ·OH radicals which facilitate the oxidation of TMB into a blue color product. In this work, the findings demonstrate that the production of H_2_O_2_ from the catalytic reaction of H_2_O_2_ accelerated by Cu^2+^ is the key factor for the oxidation of TMB in this work.

$$\eqalign{ & {{\rm{H}}_2}{{\rm{O}}_2} + {\rm{TMB}}\mathop \to \limits^{C{u^{2 + }}} {{\rm{H}}_2}{\rm{O}} \cr & + {\rm{oxidizedTMB}}\,\,\left( {{\rm{blue color}}} \right) \cr}$$

### Analytical performance of the sensitive PVA/TMB NFs toward H_2_O_2_

#### Visual detection of H_2_O_2_ by naked eye

**Fig. 5:**
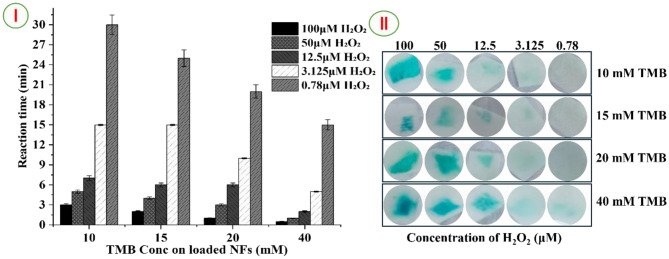
(**I**) comparison between four PVA/TMB NFs loaded with different concentrations of TMB (40, 20, 15 and 10 mM) versus reaction time (min); (**II**) color scale developed for H₂O₂ detection

The study of colorimetric response of the flexible PVA/TMB NFs to varied concentrations of H_2_O_2_ has been conducted as explained in Fig. 5I and II. In all PVA/TMB NFs no color observed after addition either the catalyst only or hydrogen peroxide only. For low concentration of TMB (10 mM) in PVA/TMB NFs were exposed to H_2_O_2_ at various concentrations. The change in visual color with definite intervals of incubation time (0, 3, 5, 15 up to 30 min), with highest concentration of H_2_O_2_ (100 µM) after 3 min revealed a deep blue color and with concentration 50 µM was observed blue color at time 5 min then the color intensity decreased with low concentrations of H_2_O_2_ (as 12.5, 3.125 and 0.78 µM) at time 7, 15 and up to 30 min,respectively. For the second PVA scaffold loaded with 15 mM TMB, the color change occurred more rapidly than in the previous scaffold, even at the highest concentration of H₂O₂ (100 µM). Within just 2 minutes, the color of the nanofiber strip transformed to a deep blue. In contrast, at lower concentrations of H₂O₂ (50, 12.5, 3.125, and 0.78 µM), the reaction with the nanofiber strips took significantly longer, with color changes observed at 4, 6, 15, and 25 minutes, respectively. The third scaffold, TMB concentration (20 mM) in nanofibers, when treated with the catalyst and H_2_O_2_ (100 mM) was given response after 1 minute as a deep blue on nanofiber mat, while with low concentrations (50, 25, 12.5, 3.125 and 0.78 µM) of H_2_O_2_ the color change takes longer to (3, 6, 10 and 20 min), respectively and the intensity of color decreased gradually with decreasing concentration from blue to bluish white color. Finally, the last nanofiber scaffold, with the highest concentration of TMB as a chromogenic agent (40 mM) when subjected to highest concentration of H_2_O_2_ (100 µM) the color changes to intense deep blue that occurred immediately after only 30 seconds. However, with low concentrations (50, 12.5, 3.125 and 0.78 µM) of H_2_O_2_ the response time becomes (1,2,5 and 15 min), respectively. It is evident that the sensing performance of the PVA/TMB NFs in the last scaffold, which contains the highest concentration of TMB, is significantly better at shorter exposure time compared to the other scaffolds. Additionally, the color intensity depends on the concentration of H₂O₂.

#### Detection of H_2_O_2_ by UV spectrophotometric method

**Fig. 6:**
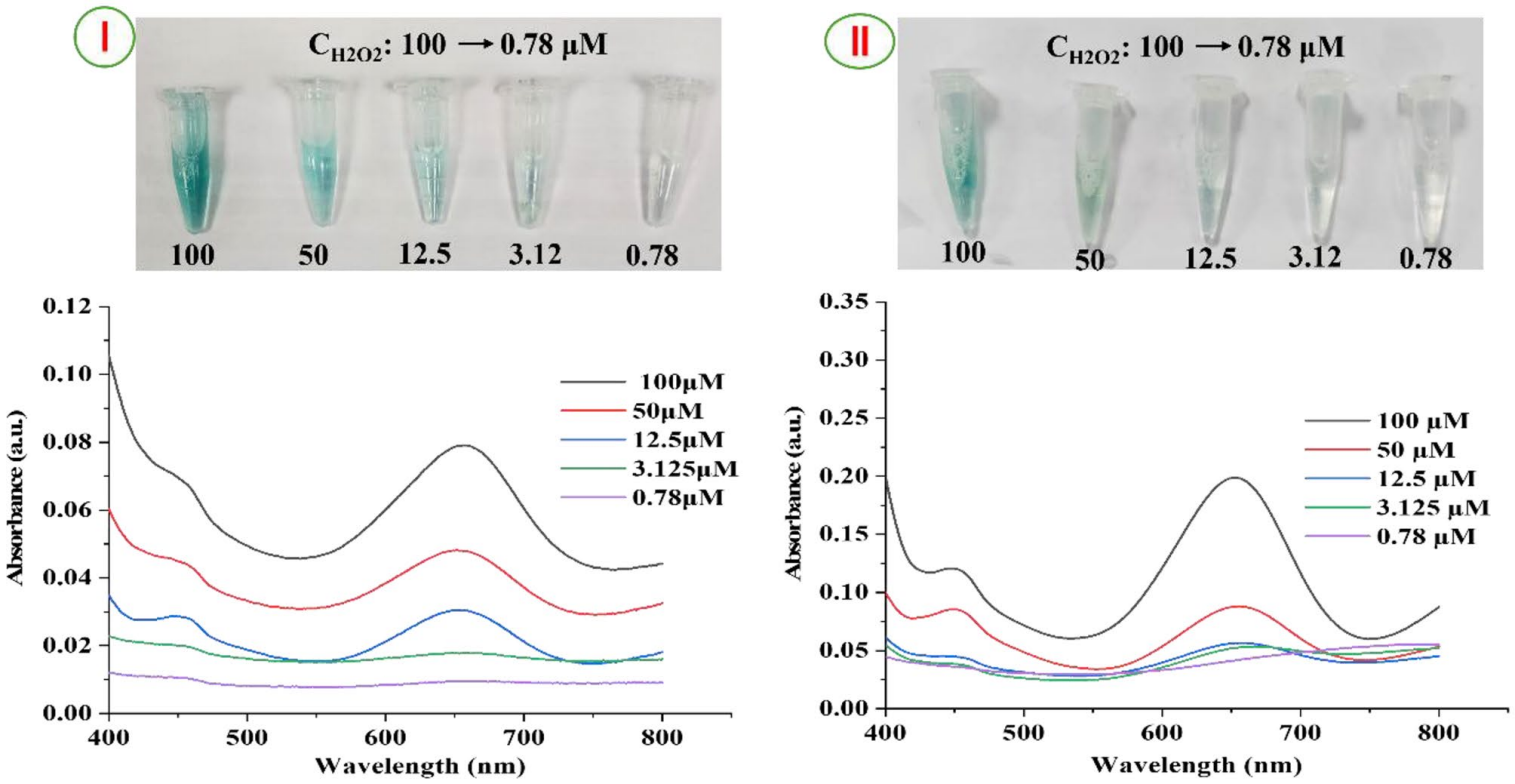
UV -Vis response of PVA/TMB NFs and Cu^2+^ as catalyst upon increase of concentration of H_2_O_2_ from 0.78 µM to 100 µM. Inset is actual photo of various levels of H_2_O_2_. (**I**) for PVA/40 mM TMB NFs and (**II**) for PVA/20 mM TMB NFs scaffold

A colorimetric assay utilizing Cu^2 +^ and PVA/TMB nanofibers was developed to determine the concentration of H₂O₂. Initially, various amounts of H₂O₂ were added to an Eppendorf tube comprising PVA/TMB nanofiber strips along with 5 µL of Cu^2 +^ (40 mM). In this experiment, we selected two PVA/TMB nanofiber scaffolds (20 mM and 40 mM), which provided the most applicable spectra and results, as illustrated in Fig. 6I and II. Hydrogen peroxide is reduced by Cu (II), resulting in generation of oxygen and water. During this enzymatic reaction, electrons are released and accepted by the chromogenic substrate, specifically the PVA/TMB nanofibers (NFs). Upon accepting these electrons, the dye-loaded nanofibers undergo oxidation, changing into a blue product that exhibits peak at 655 nm. As the concentration of H₂O₂ increased, the system’s color transitioned from colorless to blue. This change was further validated by measurements taken with a UV-Vis spectrophotometer. Notably, with raising concentrations of H₂O₂ and the corresponding increase in dye content (TMB) within the scaffolds, a considerable enhancement in absorption intensity was observed at 655 nm [33, 58].

### The Effect of pH against sensitivity of nanofiber-based sensor

**Fig. 7:**
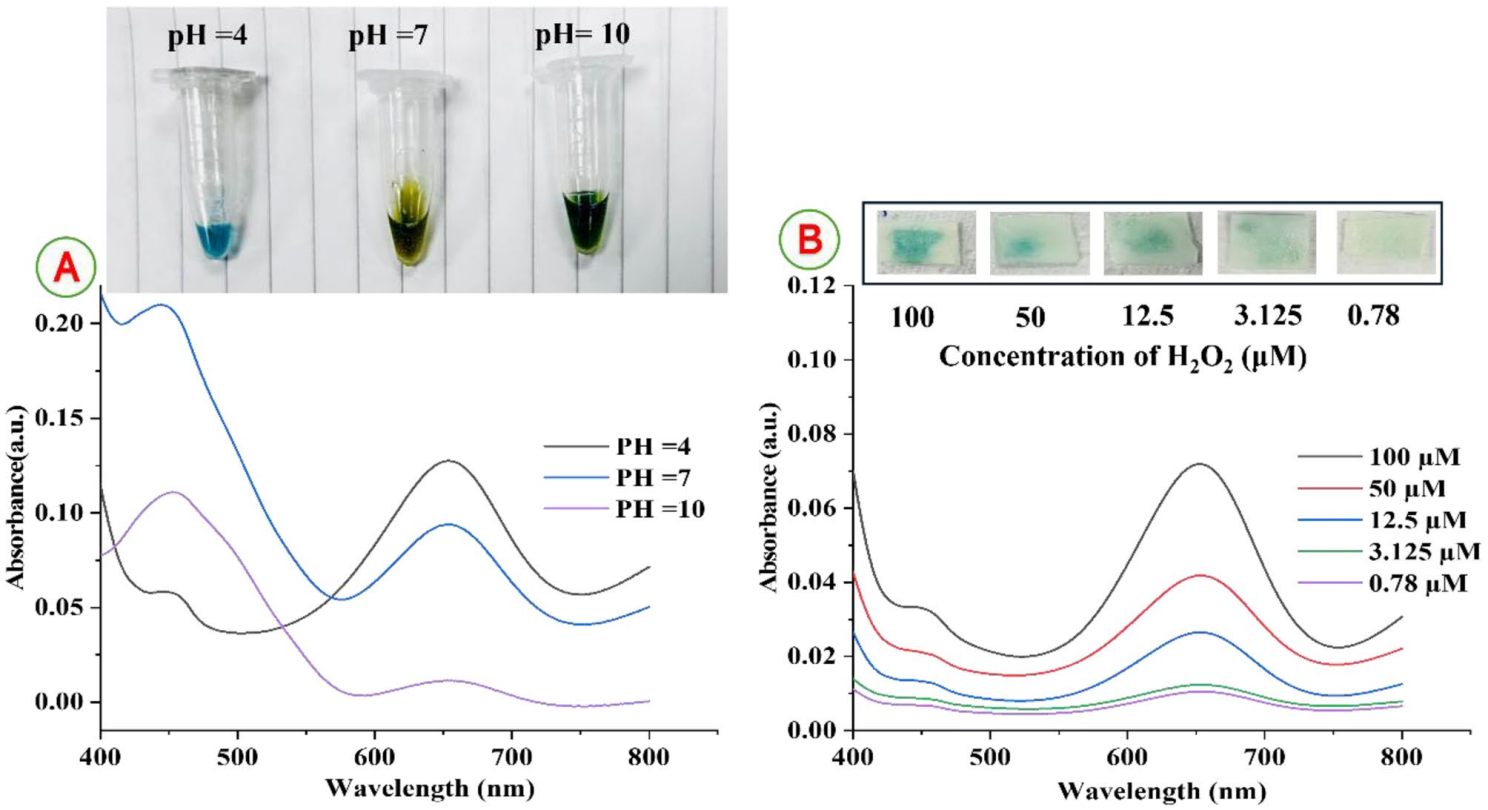
(**A**) UV-vis absorption curves of the peroxidase-like catalytic activity of Cu^2+^ ions in acetate buffer solution with varied pH values. (**B**) UV-vis absorption curves of reproducibility and stability for detection of H₂O₂ using PVA/40 mM TMB NFs after storage for 12 months (inset color scale developed for various H₂O₂ concentrations)

The catalytic activity of Cu^2+^ ions is strongly dependent on pH, like HRP or other acting as peroxidase mimetics [24]. So, the effect of different pH values of acetate buffer was examined. Thus, the sensitivity of the proposed approach tended to increase quickly as the pH of acetate buffer rose to 4.0 then declined rapidly when pH was more than 4.0, as observed in Fig. 7A. In the weakly acidic medium, fast formation of ·OH was favorable but in a strongly acid medium H_2_O_2_ converted to H_3_O_2_^+^ (oxonium ion) as seen in equation-1, which leads to decrease ·OH generation. The process of generation of O˙_2_^-^ forms in neutral and alkaline media called the sonolysis of water as illustrated in equation-2, the analytical signals reduce because ·OH in media can also react rapidly with O˙_2_^-^ to hydroxyl ions and oxygen as observed in equation-3 [59]. Wherefore, Cu^2+^ ions had maximum catalytic efficiency at pH 4.0 under slightly acidic conditions than neutral and alkaline conditions as has also been observed for Ag_3_PO_4_ nanocrystals [60], and silver nanoparticles (AgNPs) [59] which closely matches what was observed with Cu^2+^ ions as peroxidase like activity. Therefore, the ideal reaction setting for examining catalytic activity is a pH of 4.0 and was selected to detect H_2_O_2_ [24, 61].

1 $${{\rm{H}}_2}{{\rm{O}}_{2{\rm{ }}}} + {\rm{ }}{{\rm{H}}^ + }{\rm{ }} \to {\rm{ }}{{\rm{H}}_3}{{\rm{O}}^ + }$$

2 $${\rm{OH}} + {H_2}{O_{2 }} \to O_2^{ - } + {H_2}{O_2} + {H^ + } $$

3 $${\rm{O}}_2^{ - } + {\rm{ }}\cdot{\rm{OH}} \to {\rm{O}}{{\rm{H}}^ - } + {\rm{ }}{{\rm{O}}_2}$$

### Reproducibility and stability of the PVA/TMB nanofibers test strip toward H₂O₂

The results indicated that the time and color intensity were astonishingly steady with no significant alterations even after 12 months of storage as noticed in Fig. 7B. These observations demonstrate that the produced PVA/40 mM TMB NFs strip retains a similar color when exposed to hydrogen peroxide after 12 months of storage, with only a slight change in the color difference relative to freshly prepared strips. Exhibit exceptional and consistent sensing behavior. Based upon these observations, the PVA/TMB nanofibrous-based strips show promising candidature for applications in chemical sensing, other metal ions and prolonged shelf-life [62, 63].

### Linearity and detection limit

**Fig. 8:**
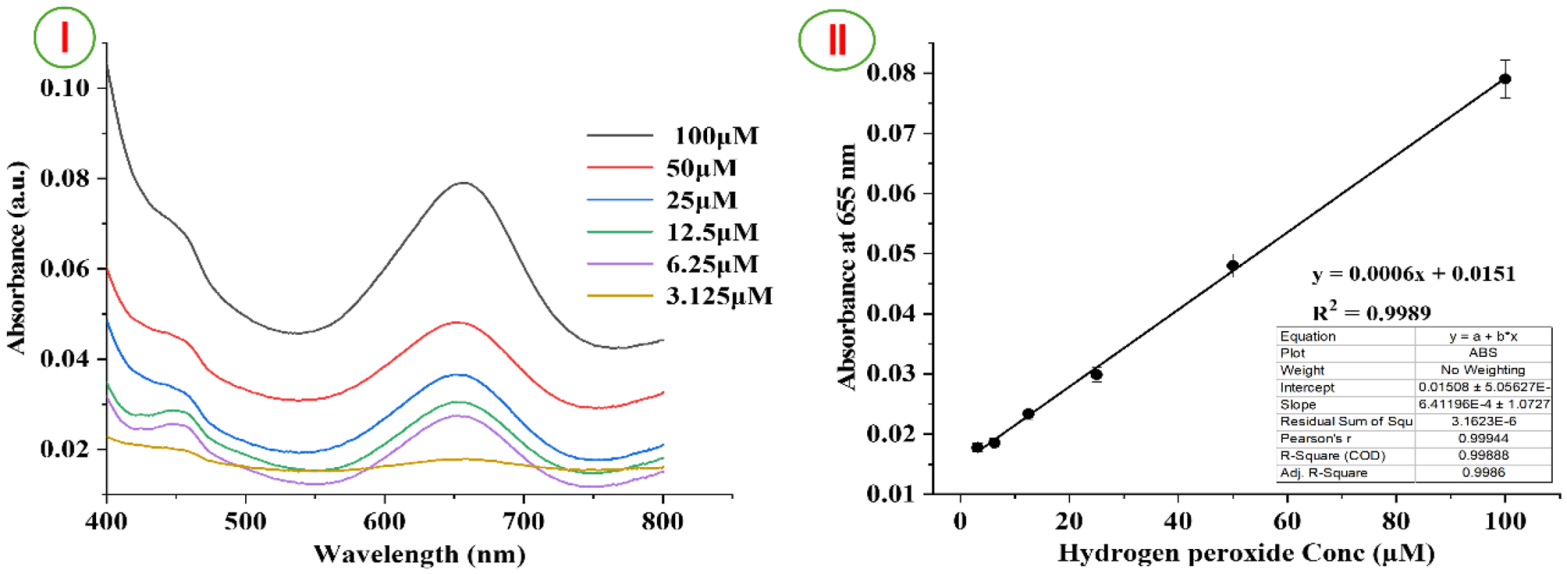
(**I**) UV–vis spectra of hydrogen peroxide sensor-based NFs with various H_2_O_2_ concentrations; (**II**) calibration curve for H_2_O_2_ detection. Experimental conditions were described in the text

The sensor’s sensitivity and dynamic measuring range were evaluated under optimum conditions using standard H₂O₂ solutions with oxidized TMB as a colorimetric indicator. Figure 8 shows that the absorbance at 655 nm increased with the concentration of H₂O₂, ranging from 0.78 to 100 µM. This indicates that the oxidation of TMB by H₂O₂ is directly dependent on its concentration. The change in absorption intensity of oxidized TMB was utilized for quantify H₂O₂ levels. Figure 8.I presents a typical concentration-absorbance curve for H₂O₂, demonstrating a steady increase in absorbance at 655 nm with increasing H₂O₂ concentration. A satisfactory linear relationship was established: A = 0.0006C + 0.0151 (µM) for H₂O₂ concentrations ranging from 3.125 to 100 µM (R^2^ = 0.9989), as shown in Fig. 8II. The limit of detection (LOD) for H₂O₂ was calculated using a signal-to-noise ratio of 3 (3δ/S), where δ represents the standard deviation of blank data and S indicates the slope of the linear equation. The detection limit was determined as follows: LOD = 3.3 (5.05627/6.4119) = 2.60 µM. Thus, the proposed sensor based PVA/40 mM TMB NFs can detect H₂O₂ with a limit of 2.60 µM, which is comparable to previously reported methods (Table 2). The linear range of the sensor extends from 3.125 to 100 µM, and the LOD of 2.60 µM is lower than the concentration of hydrogen peroxide typically found in malignant tumor cells (exceeding 50 µM). Therefore, the developed colorimetric H₂O₂ sensor holds significant potential for daily testing applications.

**Table 2:** Comparing the analytical performances of different H_2_O_2_ colorimetric sensors

Materials	Morphology	Detection output	Linear Range	LOD	Reference
Co-N doped Carbon	Nanofibers	Colorimetric	0.025–1.25 mM	40.5 μM	[64]
TA-AuNPs-3/I^-^	Nanoparticles	Colorimetric	150 and 275 μM	7.33 M	[65]
CeO_2_/Co_3_O_4_ HNs	Hollow Nanocubes	Colorimetric	0 to 7 mM	3.56 μM	[66]
Starch-SAgNPs	Nanoparticles	Colorimetric	0.45–121 μM	3.70 μM	[67]
Red-AgNPrs	Nanoprisms	Colorimetric	10–80 μM	6.19 μM	[68]
VO_2_ NFs/TMB	Nanofibers	Colorimetric	25–10000 μM	18 μM	[61]
PVA/40 mM TMB NFs catalyzed by Cu^2+^ ions	Nanofibers	Colorimetric	3.125–100 μM	2.60 μM	This work

### Selectively and specificity of PVA/TMB NFs towards H_2_O_2_

**Fig. 9:**
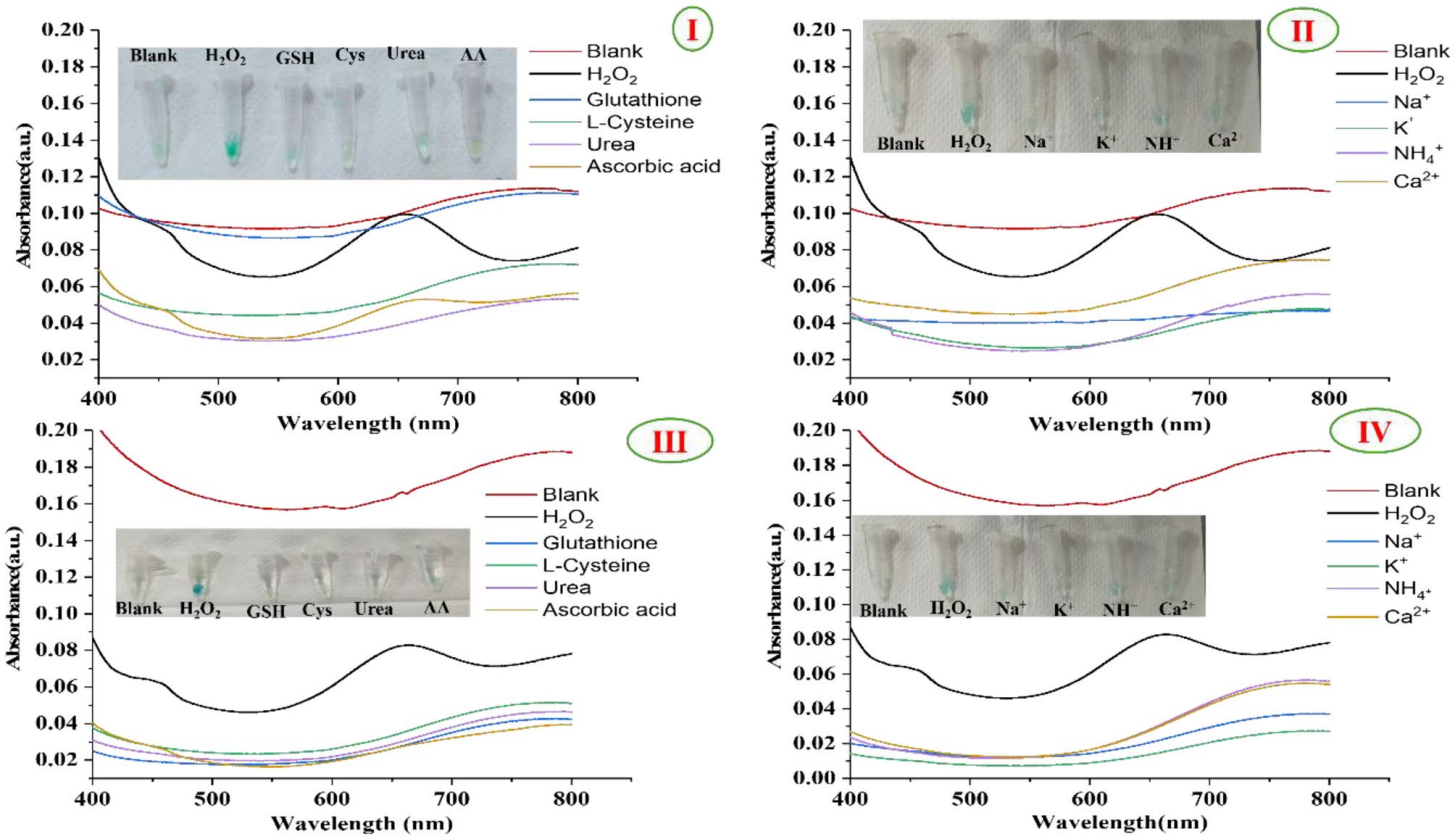
Selectivity analysis at 650 nm for detecting H_2_O_2_ over biomolecules (GSH, L-Cys, urea, AA) and ions (Na^+^, K^+^, NH_4_^+^, Ca^2+^) between 0 and 10 minutes. Biomolecules and ions are at concentration 100 μM, while H_2_O_2_ is at 10 μM. I and II use a PVA/40 mM TMB NFs scaffold while III and IV use PVA/20 mM TMB NFs scaffold. The color images describe the reaction solutions after 10 minutes

The selectivity and specificity of the colorimetric sensor based on nanofibers (NFs) for H₂O₂ were evaluated in the presence of common biological molecules found in human urine. These included GSH, L-Cys, urea, AA, and four types of ions: Na^+^, K^+^, NH₄^+^, and Ca^2 +^, all at a preset concentration of 100 µM. The PVA/TMB NFs exhibit color change under exposure to H_2_O_2_, whereas no noticeable significant change in color of NF sheets was observed for other common biological interferents and through the absorption spectra, it is found that only H_2_O_2_ with the PVA/TMB NFs produces the corresponding absorption peak in 655 nm, while others do not produce a significant absorption peak. This shows that the prepared PVA/TMB NFs have obvious catalytic ability only for H_2_O_2_ and show good selectivity specificity as illustrated in Fig. 9. Additionally, ROS (superoxides and hydroxyl radicals), and reactive nitrogen species (peroxynitrites and peroxynitrates) exist in several physiologically relevant forms. Superoxide dismutase (SOD) is an enzyme that quickly converts superoxide free radicals into hydrogen peroxide. Therefore, compared to other ROS, hydrogen peroxide is more stable and has a longer biological lifespan [69].

### Real sample sensing

As discussed in previous sections, the PVA/40 mM TMB NFs system displayed excellent and wide linear response and high selectivity toward H_2_O_2_, demonstrating superior capabilities for real-sample analysis. Measurement of urinary H_2_O_2_ offers significant clinical and diagnostic data as it is a valid biomarker of oxidative stress, a screening tool for exposure to environmental and occupational hazards. To assess the analytical efficacy of our proposed sensor in biological fluids, we performed standard addition procedures using urine samples obtained from healthy adult participants. In the unspiked urine specimens, H_2_O_2_ concentrations remained continuously below the method’s detection limit (LOD: 2.60 μM), with all measurements produced absorbances indistinguishable from the blank controls. Given this unmeasurable baseline, recovery values were determined by direct comparison between the measured H_2_O_2_ concentrations in spiked samples and the known amounts of added standard, using the following formula: Recovery (%) = (Measured spiked/Added standard) × 100. Across all tested concentrations, recoveries ranged from 96% to 103% with all relative standard deviations (RSD) values below 3.2% (Table 3) [70]. In healthy people, H_2_O_2_ concentrations in urine are normally maintained at relatively low levels, but oxidative stress processes in pathological conditions like cancer or inflammation can dramatically raise these levels [63]. Our improved colorimetric technique, PVA/40 mM TMB NFs strip-based sensor, demonstrates excellent naked eye potential by providing simple, rapid and economical identification of pathological H_2_O_2_ changes within the 3.125–100 µM range, suitable for early detection of serious diseases, diagnostic applications, treatment monitoring. The method’s reliability for H_2_O_2_ quantification in complex biological metrics is confirmed by these strong validation results, especially for applications involving clinical urine analysis.

**Table 3:** Determination of urinary H_2_O_2_ (*n* = 3)

Sample^*^	Standard added (μM)	Total found (μM)	Recovery (%)	R.S.D. (%)
Urine 1	5	5.15	103	1.6
Urine 2	10	10.7	100.7	2.345
Urine 3	50	48.3	96.68	3.2
Urine 4	100	99.60	99.60	1.5

^*^ Quantification of urine samples by diluted 10 times

### Conclusions

In this study, we successfully developed and characterized electrospun nanofiber mats composed of tetramethylbenzidine (TMB) and polyvinyl alcohol (PVA) for the creation of test strips aimed at the visual detection of hydrogen peroxide (H₂O₂). By carefully controlling the electrospinning parameters, we produced reproducible nanofibers with nanoscale dimensions, leading to an effective TMB loaded nanofiber scaffold. The experiments demonstrated that TMB, dissolved in a defined volume of DMF (5 μL), could be effectively dispersed in various concentrations (10, 15, 20, and 40 mM) within the PVA solution. The TMB-loaded nanofibers showed a uniform distribution of TMB, with the highest concentration (40 mM) yielding the most significant color response and rapid development. This concentration was thus selected for further experiments. The performance of the H₂O₂ sensor was notable, exhibiting a fast response time, high sensitivity, excellent selectivity, reproducibility, and long-term stability. It operates effectively within a linear range of 3.125 μM to 100 μM, with a low detection limit of 2.60 μM. The simplicity and ease of use of this method eliminate the need for sophisticated instrumentation or extensive sample preparation.

However, we acknowledge that while the PVA/TMB nanofiber-based sensor assay provides a reliable optical method for detecting urinary H₂O₂, it may encounter interference from various substances present in complex biological matrices, which could potentially compromise selectivity and accuracy.

Future research should prioritize optimizing the sensor for different environmental conditions and exploring adaptations for the detection of additional analytes. By addressing these limitations, we aim to enhance the sensor’s robustness and broaden its applications in biosensing, medical diagnostics, and environmental monitoring.

## Data Availability

The data sets used and analyzed during the current study are available from the corresponding author on reasonable request.
